# ASO Author Reflections: Improving Postoperative Outcomes in Colorectal Cancer Surgery: Where are We with Prehabilitation?

**DOI:** 10.1245/s10434-024-15835-3

**Published:** 2024-07-11

**Authors:** Daniel Steffens, Michael J. Solomon, Sandy Jack, Malcolm A. West

**Affiliations:** 1 Surgical Outcomes Research Centre (SOuRCe), Royal Prince Alfred Hospital, Sydney, NSW Australia; 2 Faculty of Medicine and Health, Central Clinical School, The University of Sydney, Sydney, Australia; 3 National Institute for Health and Social Care Research, Southampton Biomedical Research Centre, Perioperative and Critical Care Theme, University Hospitals Southampton, Southampton, UK; 4 Cancer Sciences, Faculty of Medicine, University of Southampton, Southampton, UK

## Past

Despite the development of minimally invasive surgical techniques and the implementation of comprehensive Enhanced Recovery After Surgery (ERAS) protocols, colorectal cancer surgery still carries significant postoperative morbidity, which slows recovery and increases healthcare costs.^1^ Prehabilitation, including exercise, nutrition and psychological interventions, has been proposed to optimize preoperative patient health and reduce postoperative morbidity;^2^ however, the effectiveness of different prehabilitation modalities in colorectal cancer surgery remains unclear.^3^ Our systematic review and meta-analysis aimed to synthesize the evidence from the latest randomized controlled trials on the impact of prehabilitation on postoperative complications and length of hospital stay in this population.

## Present

Our systematic review identified a total of 23 prehabilitation trials in colorectal cancer patients undergoing surgery.^4^ We found moderate-quality evidence that preoperative nutrition interventions reduced postoperative infectious complications by 35% and length of hospital stay by approximately 1 day compared with usual care. This highlights nutrition optimization as a promising strategy to improve surgical outcomes. However, the evidence was less certain for multimodal, exercise, and psychological prehabilitation due to the limited number of trials, variation in reported outcomes, and low quality of evidence overall. High-quality research is still needed to determine the optimal prehabilitation approach.

## Future

To advance the field of prehabilitation in colorectal cancer surgery, several key steps are necessary. First, establishing a core set of outcomes and developing prehabilitation guidelines will provide a framework for designing high-quality trials that can be meaningfully compared and synthesized. Second, investigating the impact of prehabilitation adherence on outcomes and exploring the potential synergistic benefits of multimodal interventions will help optimize prehabilitation strategies. Identifying patient factors that predict response to prehabilitation will enable personalized interventions to be targeted to those most likely to benefit. Furthermore, developing less resource-intensive prehabilitation programs that can be widely implemented is crucial for maximizing accessibility and cost effectiveness. Ultimately, appropriately powered multicenter trials incorporating these elements are required to provide definitive evidence to support the integration of prehabilitation into standard preoperative care. By reducing postoperative complications and accelerating recovery of daily activities, effective prehabilitation has the potential to significantly improve long-term patient outcomes and quality of life after colorectal cancer surgery.
